# Role of combined therapy of intravitreal Ranibizumab and topical Dorzolamide in the Treatment of foveal schisis maculopathy: A case report and review of literature

**DOI:** 10.22336/rjo.2026.62

**Published:** 2026

**Authors:** Anuradha Raj, Sahil Jain, Anurag Singla, Easther Dazine

**Affiliations:** 1 Department of Ophthalmology, All India Institute of Medical Sciences, Bathinda, Punjab, India

**Keywords:** Foveal schisis, anti-vascular endothelial growth factor, dorzolamide, XLRS = X-linked foveal retinoschisis, BCVA = Best-corrected visual acuity, Anti-VEGF = Anti-vascular endothelial growth factor, OCT = Optical coherence tomography, CAI = Carbonic anhydrase inhibitors, RPE = Retinal pigment epithelium

## Abstract

**Objective:**

Description of unusual management of the foveal schisis maculopathy.

**Methods:**

Case report and review of literature.

**Results:**

We report the case of a 25-year-old man who presented with a gradual, painless diminution of vision that persisted for 3 months. The best-corrected visual acuity (BCVA) was 20/40 and 20/120 in the right and left eyes, respectively. Fundus examination revealed bilateral spoke-wheel pattern maculopathy, and optical coherence tomography (OCT) confirmed schisis at the inner nuclear and outer plexiform layers. An important morphological and functional improvement resulted from sequential bilateral intravitreal anti-VEGF injections (0.3 mg Ranibizumab), one in each eye, followed by topical 2% dorzolamide eye drops, which helped to stabilize the disease. In this case, anti-VEGF treatment followed by topical dorzolamide demonstrated anatomical and functional improvement. This combined therapy stabilized the condition, suggesting its efficacy.

**Discussion:**

X-linked foveal retinoschisis (XLRS) is a hereditary juvenile retinal degeneration that presents with mild to moderate bilateral vision loss, with a spoke-wheel pattern on fundoscopy. As of now, there is no established effective treatment for XLRS; however, carbonic anhydrase inhibitors (CAIs) have demonstrated efficacy in reducing schisis cavities by absorption through the retinal pigment epithelium. The role of anti-VEGF therapy in treating X-linked retinoschisis (XLRS) is also limited and may offer temporary improvements in foveal retinoschisis.

**Conclusion:**

In foveal schisis maculopathy, combination therapy with anti-VEGF and topical dorzolamide may be considered. This therapy offers notable improvements in foveal retinoschisis by restoring macular architecture and serves as a maintenance treatment. Further research with larger cohorts is needed to optimize therapy regimens.

## Introduction

X-linked foveal retinoschisis (XLRS) is a prevalent degenerative retinopathy with X-linked recessive inheritance. Its prevalence ranges between 1:5000 and 1:20,0001 and is caused by mutations in the RS1 gene. It usually presents with mild to moderate bilateral vision loss in males, with a spoke-wheel pattern on fundoscopy [1]. In XLRS, visual acuity can remain stable over time; however, an accelerated decline in vision associated with central retinal atrophy typically begins in the fourth or fifth decades of life [2-4]. This article highlights the role of combined therapy with intravitreal Ranibizumab and topical dorzolamide in treating this condition in a patient who presented with foveal retinoschisis.

## Methods

We report a case of foveal schisis that responded well to the combined therapy of intravitreal Ranibizumab and topical dorzolamide.

## Results

A 25-year-old male presented to us with a history of painless, non-progressive diminution of vision in both eyes, with the left eye affected more than the right. The patient reported no history of systemic illness or chronic medication use. On examination, the best corrected visual acuity (BCVA) in the right and left eyes was 20/40 and 20/120, respectively, with a refractive error of 1Dioptre (D) sphere in each eye. The anterior segment findings were unremarkable. On dilated fundus examination, a spoke-wheel pattern over the macular region with foveal cystoid cystic spaces was noted (**Fig. 1A, B**). The central macular thickness was 477 and 545 μm for the right and left eye, respectively. On performing optical coherence tomography (OCT) raster scan through the macula, intra-retinal cystoid cystic spaces at the level of inner nuclear and outer plexiform layers were evident with no signs of macular hole or retinal detachment (**Fig. 2A, B**). The fundus fluorescein angiography showed no leakage in the macular area. This typical retinal appearance, along with the absence of a history of trauma to the eye or any inflammatory changes in an otherwise healthy young male, led to the diagnosis of foveal schisis-like maculopathy.

**Fig. 1:**
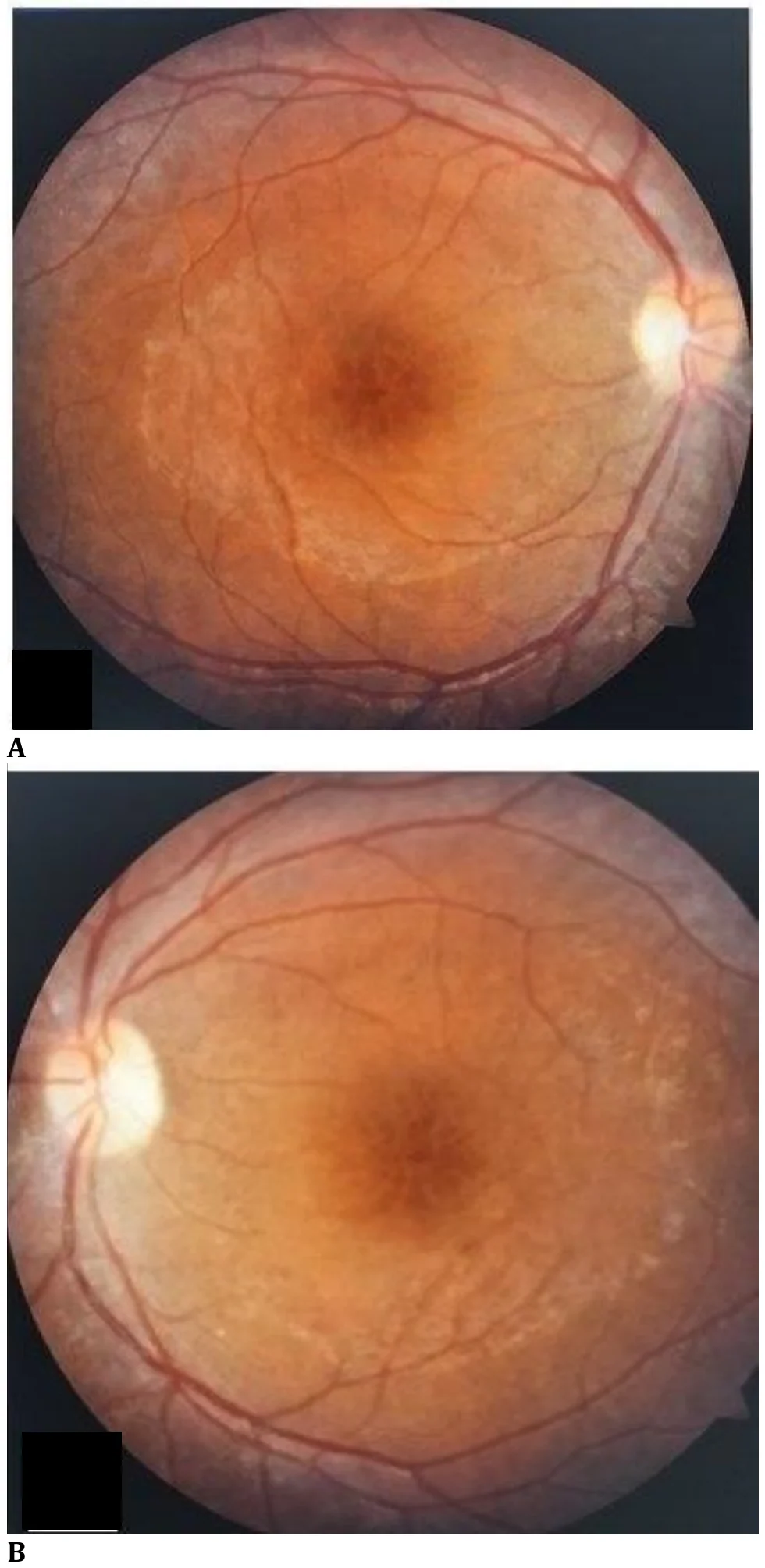
**A, B** Demonstrate a spoke-wheel pattern over the macular region with foveal cystoid cystic spaces on fundus examination

**Fig. 2:**
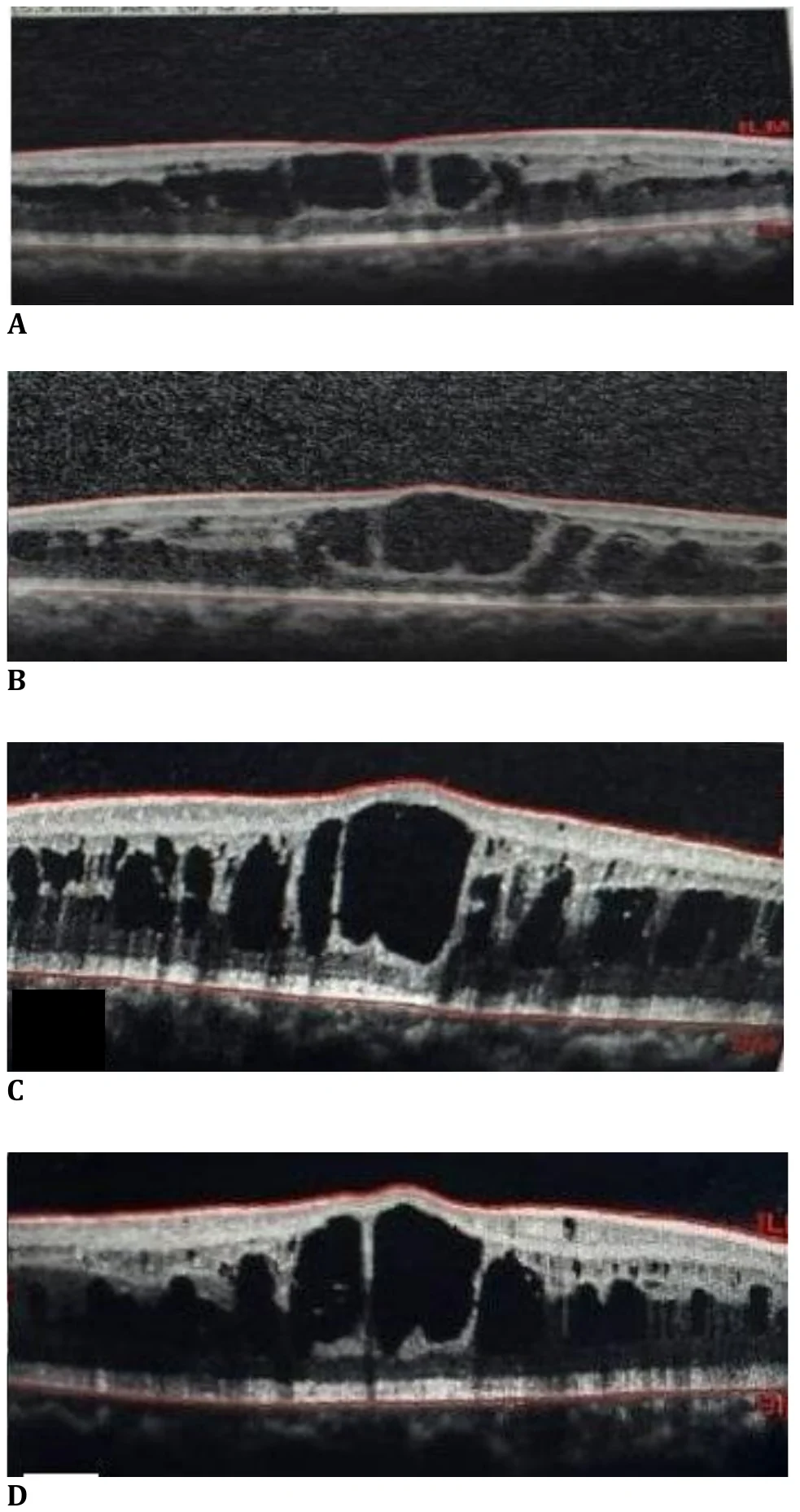
**A, B** Cystic spaces in the macular area at presentation; **C, D** Fluid-filled cystic spaces drastically increased in size after starting topical dorzolamide

The patient was started on topical Dorzolamide (2%) thrice daily in both eyes. On subsequent follow-up, BCVA worsened in both eyes, and OCT of the macula showed an increase in cystic spaces, with CMT at 641 and 719 µm (**Fig. 2C, D**). Following this, the patient was offered pars plana vitrectomy (PPV), but he declined. Then there was the option of intravitreal anti-vascular endothelial growth factor (VEGF) therapy, which the patient requested. The patient then received intravitreal ranibizumab (0.3 mg) injections in each eye at 1-week intervals. Informed consent was obtained from the patient before involving him in this manuscript.

Two-week follow-up after the second injection showed a drastic improvement in vision, reaching 20/30 and 20/80 in the right and left eyes, respectively, along with a reduction in central macular thickness on OCT to 449 and 421 μm. However, at 1-month follow-up, vision had again decreased to 20/40 and 20/120 in the right and left eyes, respectively, with increased retinal cystic spaces. The patient was then started on eyedrop dorzolamide (2%) thrice daily in both eyes. Subsequent follow-ups after three months showed maintained BCVA of 20/30 and 20/40 and stable OCT macula findings (**Fig. 3 A-D**). The patient was advised to remain on regular follow-ups.

**Fig. 3:**
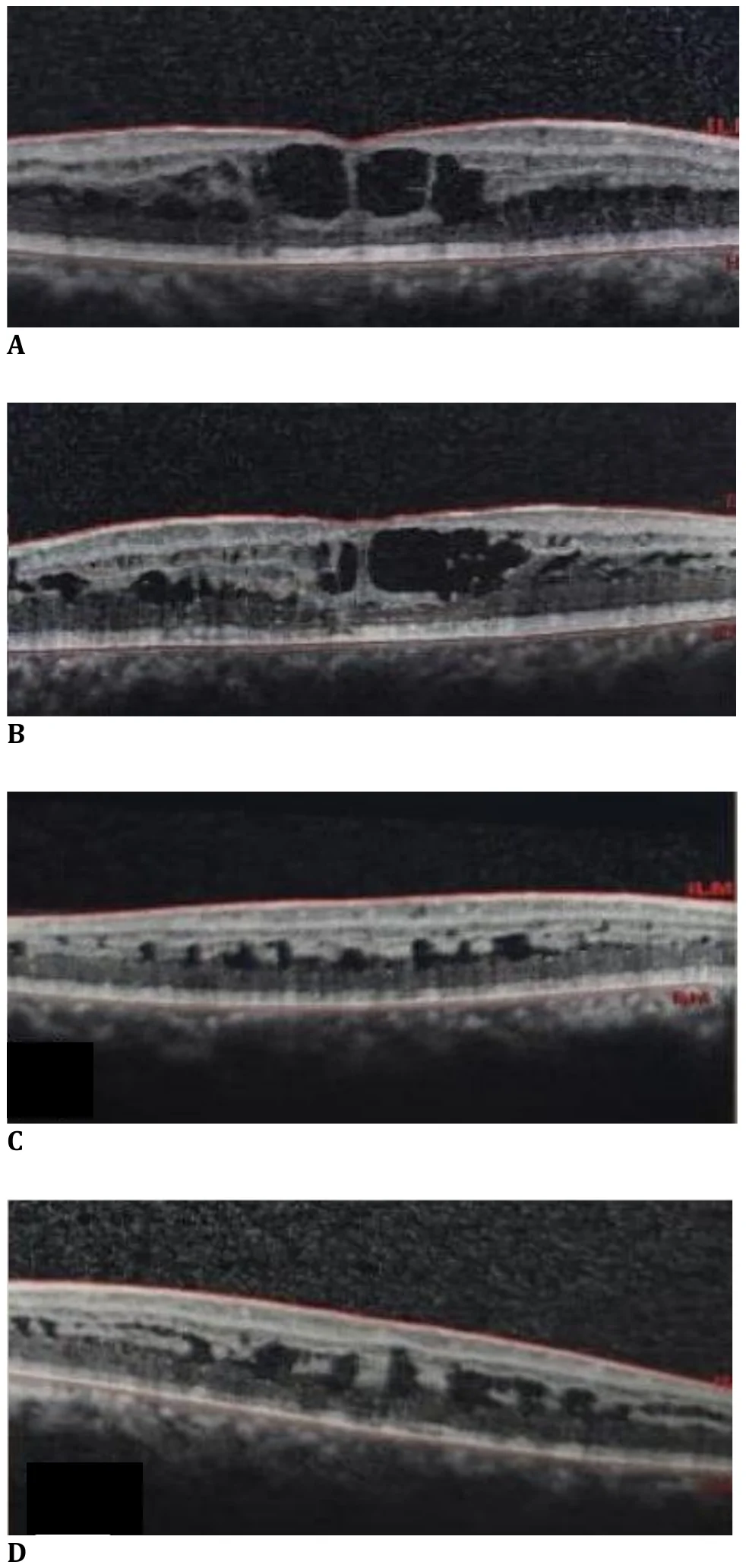
**A, B** Cystic spaces in the macular area after one month of intravitreal anti-VEGF injection in both eyes; **C, D** Flattened cystic spaces with irregular inner retinal layers after three months of starting topical dorzolamide

## Discussion

XLRS is the most common cause of macular degeneration in young males. Foveal schisis is present in all XLRS patients, with visual impairment and disturbances ranging widely based on the extent of their pathology. XLRS is a hereditary juvenile retinal degeneration marked by progressive and lifelong ocular changes.

Additionally, patients with XLRS are at an elevated risk of developing vision-compromising conditions, including retinal detachment, vitreous haemorrhage and neovascular glaucoma [5].

It typically presents with mild to moderate bilateral vision loss in males, characterized by a spoke-wheel pattern on fundoscopy, as seen in this case.

The pathophysiological basis for the formation of intra-retinal cystic spaces is a defect of retinoschisin, a protein encoded by the RS1 gene. It is expressed in bipolar and photoreceptor cells and secreted into the surrounding extracellular space, where it is thought to be essential for cell-to-cell adhesion and signal transduction between bipolar cells and photoreceptors [2,6,7]. In XLRS, a missense mutation in the RS1 gene causes the intracellular retention and degradation of retinoschisin, resulting in intraretinal cystic spaces [8]. As of now, there is no established effective treatment for XLRS; however, carbonic anhydrase inhibitors (CAIs) have demonstrated success in reducing schisis cavities in certain patients [9,10]. The mechanism underlying the reduction in cystic spaces has been hypothesized to involve the drug's absorption of soluble intraretinal retinoschisin, with assistance from the retinal pigment epithelium (RPE). Further, it is thought that CAIs promote better adhesion between the neurosensory retina and the RPE [11]. Certain authors have promoted an On-Off regimen of CAIs to allow recovery of RPE pumps, thereby facilitating treatment response with the same agent [12]. However, treatment effectiveness likely varies with the specific pathological stage of the condition. In persistent cases with irreversible retinal damage, patients become unresponsive to CAIs [10]. Lastly, factors such as dosage, route of administration, and treatment duration may influence treatment response [11]. Although anti-VEGF therapy is effective for numerous retinal diseases, its use to treat X-linked retinoschisis (XLRS) is limited [13]. Anti-VEGF treatment may offer temporary improvements in foveal retinoschisis.

The surgical treatment for XLRS remains multifactorial and physician-dependent. While pars plana vitrectomy (PPV) is usually reserved for schisis-related retinal detachment (RD), its utilization in the absence of RD has been explored. There are reports of the resolution of macular retinoschisis following PPV with internal limiting membrane peeling [14,15]. Regression of cystic spaces after PPV with internal tamponade or after scleral buckling has also been reported, but with unsatisfactory long-term outcomes [16,17].

Postoperative complications remain the major hindrance to the surgical management of XLRS. Due to the tight adhesion of the posterior hyaloid to the retina in young individuals, the most common age group affected by XLRS, there is a higher likelihood of incomplete separation, which elevates the risk of proliferative vitreoretinopathy and RD in the postoperative period [18,19]. Therefore, the decision for surgical management should be individualized and well informed.

The role of gene therapy remains crucial and encouraging in the management of patients with XLRS. Experimental studies in mouse models have shown promising long-term effects following a single administration of the human RS1 gene into the eye [20,21]. However, further studies are required before gene therapy can be considered a management option.

A solitary patient cannot establish efficacy. At best, the observation is hypothesis-generating; it is not adequate for a therapeutic recommendation. A stronger conclusion requires either a prospective case series or, ideally, a controlled study; longer observation periods are needed to separate treatment effect from natural variation.

### Short and irregular follow-up

Two weeks, one month, and three months are reported, yet the paper concludes on “maintenance” benefit. XLRS often exhibits fluctuations over many months; longer observation periods are required to separate the treatment effect from natural variation.

## Conclusion

To the best of our knowledge, this is the first case report to demonstrate that combined therapy with topical dorzolamide and anti-VEGF injection can achieve a satisfactory visual outcome in foveal retinoschisis. Combined therapy with topical dorzolamide can restore macular architecture and serve as a maintenance treatment for foveal retinoschisis. Surgical intervention should be adapted to the specific cases.
